# Colistin Resistance among *Enterobacteriaceae* Isolated from Clinical Samples in Gaza Strip

**DOI:** 10.1155/2021/6634684

**Published:** 2021-04-20

**Authors:** Mohammad Qadi, Safaa Alhato, Rasha Khayyat, Abdelraouf A. Elmanama

**Affiliations:** ^1^Department of Biomedical Sciences, Faculty of Medicine and Health Sciences, An-Najah National University, P.O.Box. 7, Nablus, Palestine, State of Palestine; ^2^Department of Medical Laboratory Sciences Faculty of Health Sciences Islamic University of Gaza, Gaza Strip, Gaza, Palestine, State of Palestine

## Abstract

Bacterial infections, especially drug-resistant infections, are a major global health issue. The emergence of multidrug-resistant (MDR) strains of *Enterobacteriaceae* and the lack of new antibiotics have worrisome prospects for all of humanity. Colistin is considered the last-line drug for MDR Gram-negative bacteria (GNB), and it is often used for treatment of respiratory infections caused by MDR-GNB. In recent years, there has been a marked increase in the incidence of colistin-resistant infections. The main objective of this study was to investigate the presence of colistin resistance among clinical GNB isolated from Gaza Strip hospitals. Clinical *Enterobacteriaceae* isolates (100) were obtained from microbiology laboratories of the hospitals of different geographical locations in Gaza Strip Governorate over a period of six months. Samples were cultured, and bacterial identification was performed by standard microbiological procedures. *Enterobacteriaceae* isolates were tested for their antimicrobial susceptibility by the disk diffusion method and the MIC method for colistin. Varying degrees of susceptibility were observed for the isolates against the tested antimicrobials even within members of the same antimicrobial class. Amikacin was the most effective drug (74%), followed by chloramphenicol (48%), fosfomycin, and gentamicin (45%). High resistance was recorded against trimethoprim (85%) and tetracycline (83%). Only 59% of the tested isolates were interpreted as susceptible, while 41% was classified as resistant. The highest resistance to colistin was found to be among the *Proteus* spp. (63.2%), followed by *Serratia* spp. (57.1%). The lowest resistance was observed among *Klebsiella* isolates (31.6%). Only 39.0% of meropenem-resistant *Enterobacteriaceae* was susceptible to colistin, while 45.8% of imipenem-resistant *Enterobacteriaceae* was susceptible to colistin. The overall resistance to colistin was high (41%) among tested clinical isolates. Furthermore, 89% was MDR. These limit and complicate treatment options for the infections caused by *Enterobacteriaceae* in Gaza Strip. This calls for immediate actions to control and monitor the use of antimicrobials in general and colistin in particular.

## 1. Introduction

The global rise in the phenomenon of antimicrobial resistance in the fight against bacterial infections is very disturbing, and concerns regarding this issue are increasing, as it complicates infectious disease treatment and increases the financial burden on healthcare systems [1].

Bacterial infections, especially drug-resistant infections, are a major global health issue. The emergence of multidrug-resistant (MDR) strains of bacteria and the lack of new antibiotics have worrisome prospects for all of humanity. A recent report suggests failing to control drug-resistant infections that may cause an excess of 10 million deaths per year and may cost up to US$ 100 trillion by 2050 [2].

Colistin is considered the last-line drug for MDR Gram-negative bacteria (GNB) [3], and it is often used for treatment of respiratory infections caused by MDR-GNB. In recent years, there has been a marked increase in the incidence of colistin-resistant infections [4].

Colistin resistance is caused by decreases in the net negative charge of the outer membrane, loss of lipid A, or efflux pumps, and the most common resistance mechanism in *Enterobacteriaceae* is the covalent modification of the lipid A moiety of lipopolysaccharide (LPS) via cationic substitution; these modifications neutralize the negative charge of LPS and subsequently reduce the binding affinity of colistin for its target [5].

Increasing the use of colistin for treatment of infections caused by GNB has led to the emergence of colistin resistance in several countries worldwide. Although resistance to polymyxins is generally less than 10%, it is higher in the Mediterranean and Southeast Asia (Korea and Singapore), where colistin resistance rates are continually increasing [6].

Antimicrobial susceptibility data for 178 carbapenemase-producing *Klebsiella pneumoniae* (KPC-Kp) isolates revealed that 76 (43%) were resistant to colistin [7].

An unpublished report in Gaza showed high resistance percentages among *Escherichia coli* isolated from poultry farms. Colistin use in the poultry industry in Gaza strip is indiscriminate, and farmers use it without prescription. This may contribute to resistance to colistin among animals' bacteria which finds its way to humans through food and other means.

This study aims at investigating the resistance of *Enterobacteriaceae* bacteria to antimicrobials in general and particularly to colistin.

## 2. Materials and Methods

### 2.1. Bacterial Isolate Sources

One hundred clinical isolates (*Enterobacteriaceae*) were obtained from microbiology laboratories belonging to the Ministry of Health hospitals (20 isolates from each hospital: Al-Shifa, European Gaza Hospital (EGH), Al-Aqsa, Nasser, and Indonesian hospitals) during the period from December 2018 to May 2019. The isolates were presumptively identified by the microbiology laboratory of the corresponding hospitals and were reidentified at the Islamic University of Gaza microbiology laboratories.

### 2.2. Inclusion and Exclusion Criteria

All *Enterobacteriaceae* isolated during the study period in the mentioned hospitals were included, and no *Enterobacteriaceae* isolates were excluded.

### 2.3. Isolate Collection and Transportation

Clinical isolates were collected on a weekly basis from the five laboratories. Each lab was supplied with freshly prepared triple sugar iron agar slants (TSIA). Technicians streaked each isolate into a separate TSIA slant, incubated overnight, and placed in a refrigerator for temporary storage. Within one week of isolation, the isolates were transported to the microbiology laboratory at the Islamic university of Gaza for identity confirmation and antimicrobial testing.

### 2.4. Isolate Identification

After purification streak, each isolate was subjected to conventional biochemical tests such as TSIA, Simmons citrate, urease, methyl red, sulfide-indole-motility, and oxidase in addition to Gram staining.

### 2.5. Antimicrobial Susceptibility Testing

Each isolate was subjected to antimicrobial susceptibility testing using the disk diffusion method in accordance to the procedures and guidance of clinical laboratory sciences institute [8]. Commercial antimicrobial disks (Liofilchem, Italy) were placed onto the surface of preswabbed Muller–Hinton agar plates with a 0.5 McFarland calibrated inoculum of the test organism. Plates were placed in a refrigerator for 15 minutes to allow proper diffusion of antimicrobials and then incubated overnight at 37°C. Interpretation of the results was performed according to antimicrobial disk manufacturer tables.

### 2.6. Colistin Minimum Inhibitory Concentration (MIC)

The microbroth dilution method using the 96-microtiter plate was employed. Serial dilutions of pure colistin (Sigma-Aldrich Inc.) were tested against a standardized bacterial inoculum. After 16–20 hours of incubation, 20 ul of tetrazolium chloride was added to each well and incubated for 15 minutes. MIC was calculated based on colour development.

Because CLSI does not provide breakpoints for *Enterobacteriaceae* when testing colistin, we used the European Committee on Antimicrobial Susceptibility Testing (EUCAST) MIC breakpoints for colistin for the purpose of interpretation: ≤2 mg/l susceptible and >2 mg/*l* resistant [9].

## 3. Results

**Figure 1:**
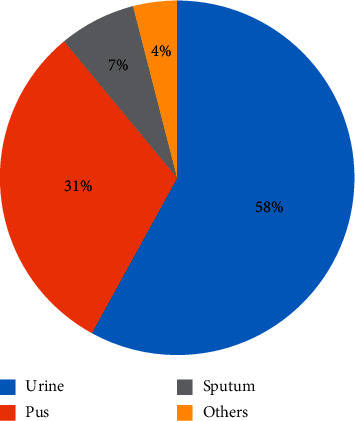
Isolates investigated in this study were obtained from sample groups (*∗*Others, 2 from blood culture and 1 each from cerebrospinal fluid and high vaginal swab). The collected *Enterobacteriaceae* consisted of *E*. *coli* (51%), *Klebsiella* spp. (19%), *Proteus* group (19%), *Serratia* spp. (7%), and *Enterobacter* spp. (4%).

The *Enterobacteriaceae* isolates used in this study were obtained from clinical samples, collected from different sources as shown in Figure 1. The pus group includes pus from different sources such as wound and ear charge samples.

The distribution of *E*. *coli*, *Klebsiella* spp., *Proteus* group, *Serratia* spp., and *Enterobacter* spp. isolates according to their source is given in Table 1.

*E*. *coli* was isolated mainly from urine samples (74.5%), followed by pus (21.6), while the *Proteus* group was isolated mainly from pus (47.4%), followed by urine samples (36.8%). In general, urine culture constituted the major source of the isolates (58%), followed by pus (31%) as given in Table 2.

### 3.1. Antimicrobial Resistance of Clinical *Enterobacteriaceae* Isolates

Varying degrees of susceptibilities were observed for the isolates against the tested antimicrobials even within members of the same antimicrobial class. Amikacin was the most effective drug with 74%, followed by chloramphenicol (48%) and fosfomycin and gentamicin (45%). High resistance was recorded against trimethoprim (85%) and tetracycline (83%). 89 isolates (89%) were MDR, as given in Table 3.

### 3.2. Colistin MIC

Bacterial isolates that showed resistance at a concentration higher than 2 mg/l were classified as resistant. Only 59% of the tested isolated were interpreted as susceptible, while 41% was classified as resistant.  Table 4 provides the MIC value obtained for 100 isolates tested against colistin sulfate.

The highest resistance to colistin was found to be among the *Proteus* group (63.2%), followed by *Serratia* (57.1%). The lowest resistance was observed among *Klebsiella* isolates (31.6%) as given in Table 5. Despite variations in resistance among the different genera, no statistical difference was detected (*P* = 0.154).

### 3.3. Colistin Resistance among Carbapenem-Resistant *Enterobacteriaceae*

Colistin is being used as last choice for carbapenem-resistant *Enterobacteriaceae*, and therefore, resistance against colistin was compared to that of imipenem and meropenem. Only 39.0% of meropenem-resistant *Enterobacteriaceae* was susceptible to colistin, while 45.8% of imipenem-resistant *Enterobacteriaceae* was susceptible to colistin as given in Table 6.

## 4. Discussion

One hundred clinical isolates of *Enterobacteriaceae* were tested against 16 antimicrobials. The lowest antimicrobial resistance was for amikacin (19%); this percent is higher than the percent reported years ago, where the percent of amikacin resistance among GNB responsible for nosocomial bacteremia was 3.9%, while among community-acquired isolates, it was 1.8% [10]. On the other hand, it is lower than amikacin resistance among ESBL-producing isolates of *Enterobacteriaceae* in a study performed by Tayh et al. in 2019 [11] which was 33.3%

The highest antimicrobial resistance was against ampicillin (89%); this percent is higher than the percent reported in 2003 [12], which was 71.6%. This means that ampicillin resistance increases with the rise of its use over time. Another study [11] conducted on ESBL-producing *Enterobacteriaceae* in urinary tract infections showed that the percent of ampicillin resistance in ESBL-producing isolates was 100%, and in non-ESBL-producing isolates, it was 58.2%. Moreover, in a study conducted on *E*.*coli* isolated from chicken droppings in Gaza strip [13], the percent of ampicillin resistance was 100%.

Our results showed that the percent of colistin resistance was 41%. Interestingly, this percent is lower than the percent reported in [14], a study which revealed a percent of 63.4% of colistin resistance and higher than the percent of colistin resistance in the study conducted on *E*. *coli* isolates [13] which was 14.5%.

Percent of *E*. *coli* resistance to colistin was 33.3%, and for *Klebsiella*, it was 31.6%.

It is obvious that we are reporting a higher percent of colistin resistance in comparison to the published data in Kuwait in 2018 by Alfoiuzan et al. [15] where the team reported resistance of 4.3% for *E*. *coli* and 7.7% for *Klebsiella*.

Carbapenem-resistant Gram-negative pathogens have become a major healthcare burden in the 21^st^ century, and treatment options had been limited to agents such as colistin and tigecycline in combination with other antibiotics [16]. In this study, only 39% of meropenem-resistant *Enterobacteriaceae* was susceptible to colistin, while 45.8% of imipenem-resistant *Enterobacteriaceae* was susceptible to colistin. Our findings highlight how much resistance to colistin has increased within the last ten years. For example, in England, ten years ago, the activity of colistin was evaluated against 81 carbapenem-resistant *Enterobacteriaceae* isolates, and colistin was active against 75/81 isolates (92.6%) [17].

In [18] a study of colistin resistance in *Klebsiella pneumoniae* and *E*.*coli* strains isolated from cancer patients, 45% of colistin-resistant isolates were meropenem resistant.

In [19] a study, the percent of imipenem and meropenem resistant GNB was 8.1% and 0.8% respectively, and in the [11] study, the percentage of imipenem resistance in ESBL-producing isolates of *Enterobacteriaceae* was 20%. Those reported percentages are much less than the percent determined by our study, which was 43.9% for imipenem and 68.3% for meropenem. This calls for setting policies to (1) prevent misuse and overuse of antibiotics in general and carbapenems, and colistin in particular, especially with the high rate of MDR detected in our study, and (2) applying procedures for infection control and screening policies for antibiotic resistance on a routine basis.

## 5. Conclusion

In conclusion, overall resistance to colistin was high (41%), and in the same context, MDR percentage was 89% among tested clinical isolates. These limit and complicate treatment options of infection caused by *Enterobacteriaceae* in Gaza Strip, which in turn calls for immediate actions to control and monitor the use of antimicrobials in general and colistin in particular.

## Figures and Tables

**Table 1:** *Enterobacteriaceae* recovered from clinical specimen distribution by hospitals.

*Enterobacteriaceae*	Hospital name					Total
	Indonesian hospital	Al-Shifa hospital	Al-Aqsa hospital	Nasser medical complex	European gaza hospital	
*E*. *coli*	8	15	11	7	10	51
	15.7%	29.4%	21.6%	13.7%	19.6%	100.0%

*Enterobacter* spp.	2	0	1	0	1	4
	50.0%	0.0%	25.0%	0.0%	25.0%	100.0%

*Proteus* group	2	1	1	11	4	19
	10.5%	5.3%	5.3%	57.9%	21.1%	100.0%

*Serratia* spp.	2	2	0	0	3	7
	28.6%	28.6%	0.0%	0.0%	42.9%	100.0%

*Klebsiella* spp.	6	2	7	2	2	19
	31.6%	10.5%	36.8%	10.5%	10.5%	100.0%

Total	20	20	20	20	20	100
	20.0%	20.0%	20.0%	20.0%	20.0%	100.0%

*P* value = 0.002.

**Table 2:** Distribution of the isolates according to clinical sample type.

Isolate	Sample type						Total
	Urine	Pus	Sputum	H.V.S	CSF	Blood	
*E*. *coli*	38	11	1	1	0	0	51
	74.5%	21.6%	2.0%	2.0%	0.0%	0.0%	100.0%

*Enterobacter* spp.	1	2	1	0	0	0	4
	25.0%	50.0%	25.0%	0.0%	0.0%	0.0%	100.0%

*Proteus* group	7	9	1	0	0	2	19
	36.8%	47.4%	5.3%	0.0%	0.0%	10.5%	100.0%

*Serratia* spp.	0	5	2	0	0	0	7
	0.0%	71.4%	28.6%	0.0%	0.0%	0.0%	100.0%

*Klebsiella* spp.	12	4	2	0	1	0	19
	63.2%	21.1%	10.5%	0.0%	5.3%	0.0%	100.0%

Total	58	31	7	1	1	2	100
	58.0%	31.0%	7.0%	1.0%	1.0%	2.0%	100.0%

*P* = 0.006.

**Table 3:** Percentage of antimicrobial resistance of *Enterobacteriaceae* tested against 15 antimicrobials.

Antimicrobial	*S*	*I*	*R*
Amikacin	74	7	19
Chloramphenicol	48	11	41
Fosfomycin	45	8	47
Gentamicin	45	6	49
Ciprofloxacin	43	5	52
Meropenem	39	10	51
Imipenem	33	22	45
Ceftriaxone	23	14	63
Ceftazidime	16	7	77
Trimethoprim	14	1	85
Tetracycline	13	4	83
Cefotaxime	12	9	79
Cefuroxime	12	6	82
Ampicillin	11	0	89
Trimethoprim-sulfamethoxazole	8	8	84

**Table 4:** MIC values for various *Enterobacteriaceae*.

MIC in mg/l	*E*. *coli*	*Enterobacter*	*Proteus* group	*Serratia*	*Klebsiella*	Total
16	6	1	8	1	2	18
	33.3%	5.6%	44.4%	5.6%	11.1%	100%

8	6	1	4	1	3	15
	40.0%	6.7%	26.7%	6.7%	20.0%	100%

4	5	0	0	2	1	8
	62.5%	0.0%	0.0%	25.0%	12.5%	100%

2	3	1	0	1	1	6
	50.0%	16.7%	0.0%	16.7%	16.7%	100%

1	9	0	3	0	2	14
	64.3%	0.0%	21.4%	0.0%	14.3%	100%

0.5	22	1	4	2	10	39
	56.4%	2.6%	10.3%	5.1%	25.6%	100%

Total	51	4	19	7	19	100
	51.0%	4.0%	19.0%	7.0%	19.0%	100%

**Table 5:** MIC values for various *Enterobacteriaceae*.

*Enterobacteriaceae*	Colistin MIC		Total
	Susceptible	Resistant	
*E*. *coli*	34	17	51
	66.7%	33.3%	100%
*Enterobacter*	2	2	4
	50.0%	50.0%	100%
*Proteus* group	7	12	19
	36.8%	63.2%	100%
*Serratia*	3	4	7
	42.9%	57.1%	100%
*Klebsiella*	13	6	19
	68.4%	31.6%	100%
Total	59	41	100
	59.0%	41.0%	100%

*P* = 0.154.

**Table 6:** Colistin resistance among carbapenem-resistant *Enterobacteriaceae*.

Colistin	Susceptible		Intermediate		Resistant		Total (%)		*P* value
Meropenem									
Susceptible	28	47.5%	8	13.5%	23	39.0%	59	59.0	0.014
Resistant	11	26.8%	2	4.9%	28	68.3%	41	41.0	

Imipenem									
Susceptible	16	27.1%	16	27.1%	27	45.8%	59	59.0	0.192
Resistant	17	41.5%	6	14.6%	18	43.9%	41	41.0	

## Data Availability

The data used to support the findings of this study are included within the article.
